# Work Engagement Among Public Employees: Antecedents and Consequences

**DOI:** 10.3389/fpsyg.2021.684495

**Published:** 2021-10-22

**Authors:** Rushana Khusanova, Seung-Wan Kang, Suk Bong Choi

**Affiliations:** ^1^College of Business, Gachon University, Seongnam, South Korea; ^2^College of Global Business, Korea University, Sejong City, South Korea

**Keywords:** job meaningfulness, work engagement, task interdependence, employee work performance, public sector

## Abstract

This study is an investigation of the relationships among job meaningfulness, work engagement, and performance, including testing for a possible mediation effect of work engagement on the relationship between job meaningfulness and performance. We examine task interdependence as a boundary condition that facilitates employee engagement using two-stage multiple-source respondent data drawn from a sample of 183 Uzbek employees from public organizations and their 47 supervisors to test the hypotheses. The research findings confirm a positive association between job meaningfulness and engagement and the relationship between work engagement and performance. Mediation analysis using bootstrapping indicated that work engagement explained the influence of meaningfulness on performance. Furthermore, task interdependence negatively moderated the relationship between meaningfulness and engagement. This study responds to calls for researchers to identify the key and situational drivers of work engagement as well as examine the importance of meaningfulness in the public sector. It also increases the external validity of the findings by examining the relationship between engagement and performance in a non-Western context, namely, Islamic Uzbekistan. Despite the limitations of this research, the empirical findings contribute to the growing body of research on work engagement and meaningfulness in public organizations.

## Introduction

In the contemporary world, an increasing number of organizations have been striving to become more sustainable because sustainability is viewed as indispensable for organizations’ competitive advantage in the marketplace (Di Fabio, 2017). Thus, the topic of organizational sustainability has received a great deal of interest from both business and academia over the past two decades (Spreitzer et al., 2012; Florea et al., 2013). To achieve sustainable development, organizations need to consider environmental, economic, and human dimensions in a comprehensive and enduring way (Hart and Milstein, 2003; Spreitzer et al., 2012; Florea et al., 2013). Although all three dimensions hold pivotal importance to long-term organizational success, the human dimension is often given less attention than the environmental and economic dimensions of organizational sustainability. The human dimension encompasses improving social health and employee well-being, and as such, employee work engagement could be a core component of the human dimension of organizational sustainability (Spreitzer et al., 2012; Florea et al., 2013; Kim et al., 2016). In Uzbekistan, psychology researchers have recently been criticized for focusing on physical illness to the exclusion of mental wellness (Ernazarov, 2020; Abdulhaevna, 2021). Researchers have suggested that organizations look at optimal functioning and the roles of positive mental state such as work engagement and supportive connections in promoting well-being; the latter is understood to be the primary focus of psychology of sustainability and sustainable development (Shimazu et al., 2010).

Employee work engagement is defined as a “positive, fulfilling, work-related state of mind that is characterized by vigor, dedication, and absorption” (Schaufeli et al., 2002a,b, p. 74). Kahn (1990) conceptualized engagement as “harnessing of organization members’ selves to their work roles” and stated that in engagement, “people employ and express themselves physically, cognitively, and emotionally during role performances” (p. 694). Demerouti et al. (2010) emphasized the benefits of work engagement for individuals and for organizations because the way individuals accomplish their work and fulfill their tasks depends on the extent to which they are engaged in their work. Rich et al. (2010) described engaged employees as more attentive and focused on their responsibilities than less engaged employees, as emotionally connected to their role tasks, and as more enthusiastic workers, and other researchers suggested that because engaged employees are also active in social activities and hobbies outside work (Schaufeli et al., 2001), positive effects of work engagement spill over into private life and vice versa (Grzywacz and Marks, 2000), which in turn leads to improved individual and group performance.

Because engaged employees possess energetic and affective connections with their work activities, see themselves as capable of dealing with job demands, and transfer their engagement to others at work (Bakker, 2009; Demerouti et al., 2010), they are more likely to contribute to sustainable individual and organizational development while promoting a healthy workplace (Bakker et al., 2011). It is crucial for organizations to sense the true essence of work engagement, especially in the public sector, to better identify its drivers (Mostafa and Abed El-Motalib, 2020). The government in Uzbekistan sees state employees as one of the main assets in promoting public sector reform (Ergashev, 2006), and thus, government employees’ work attitudes are of the utmost importance to administrators there (Ergashev, 2006). The form of ownership is the main distinctive point between state and private organizations in Uzbekistan. Specifically, the government controls and operates public sector organizations, whereas the state has no stake in private sector organizations (Ergashev, 2006). Public organizations are vulnerable to political constraints, which lead to frequent changes in policy. Their goals are pursued through political processes rather than by individual managers as in private organizations (Ernazarov, 2020). Another characteristic of public organizations is that they usually have more formal decision-making procedures, and another way they differ from the private sectors is that these organizations have few rivals in providing services such as in education and health (Ernazarov, 2020; Abdulhaevna, 2021). Although research on work engagement is flourishing, public administration scholars have given very limited attention to the antecedents of work engagement in public organizations (Andrews and Mostafa, 2019; Mostafa and Abed El-Motalib, 2020). Because disengaged employees are costly to public organizations (Mostafa and Abed El-Motalib, 2020), identifying the drivers of work engagement in that sector is important (Mostafa and Abed El-Motalib, 2020). Recent researchers have reported ethical leadership to be a key driver of work engagement in government organizations (Mostafa and Abed El-Motalib, 2020), and we propose another in this study: job meaningfulness. We suggest job meaningfulness as an underlying cause of work engagement in public organizations, and we define it as referring to the extent to which employees find their work significant and valuable (Ahonen et al., 2018). Steger and Dik (2009) observed that employees find meaning in their jobs when they clearly understand their abilities, what is expected of them, and how to successfully function in their work environments. We assume that when employees view their work as important, place higher value on work, and feel that they contribute to society through their work, they will be enthusiastic about their work, have high energy, and be so often immersed in their jobs that time flies for them.

Perry and Hondeghem (2008) argued that many employees choose to work in the public sector in anticipation of doing meaningful work and contributing to society. Hence, studying job meaningfulness is quite important in public settings (Tummers and Knies, 2013). Tummers and Knies (2013) claim that few researchers have analyzed the importance of job meaningfulness for work outcomes in public organizations specifically. Thus, filling this gap in the literature, we investigated the significant role of job meaningfulness in facilitating public sector employees’ work engagement. Moreover, Shuck et al. (2011) asserted that despite the significant role of employee engagement in work settings, there remains a shortage of academic research on situational drivers of work engagement. Responding to this call, in this study, we tested task interdependence (i.e., the extent to which individuals depend on one another to accomplish their jobs; Pinjani and Palvia, 2013) as a boundary condition on the relationship between job meaningfulness and engagement.

Several researchers have examined the direct effects of meaningfulness (Wang and Xu, 2019) and task interdependence (Lee et al., 2018) on employee engagement, but to date, none has investigated whether these two factors interact to influence employees’ work engagement. Therefore, for this study, we examined the interaction effect of job meaningfulness and task interdependence on engagement. We suggest that task interdependence at work will be more salient in promoting engagement among employees who fail to find their jobs meaningful, whereas employees who perceive high meaningfulness might not feel it necessary to work together with coworkers to invest extra effort in their work.

We also postulate the positive relationship between employee engagement and performance in this study. Engaged employees invest their emotional, cognitive, and physical energies in their work to achieve superior performance (Demerouti et al., 2010; Rich et al., 2010), and we argue that employees who are energetic, absorbed in, and dedicated to their work will exhibit high performance. Indeed, findings from many studies confirmed a significant relationship between employee engagement and performance (Schaufeli et al., 2006a,b; Bakker and Bal, 2010; Buil et al., 2019). However, researchers have mostly conducted these studies in the engagement-performance domain in Western countries, and data are insufficient from non-Western contexts (Kim, 2017; Ismail et al., 2019). Based on this limitation, we tested the positive relationship between employee engagement and performance in a non-Western context, specifically, in Islamic Uzbekistan.

Uzbekistan is a country where Muslims are a sizable majority (Ro’i, 2015). In Islam, work is one way to worship God (Marzband et al., 2016), and Muslims tend to believe they will be held accountable for their work-related attitudes and performances (Rehman, 2010). Consequently, Muslims tend to conduct their work lives with honesty and dignity. Indeed, predominantly Islamic cultures value dedication to work as a virtue (Yousef, 2000; Akdere et al., 2006). Furthermore, Uzbekistan has a collectivistic culture that emphasizes group binding and mutual obligations among individual group members (Ernazarov, 2020). Organizations in such cultures tend to be extended families, and organization-employee relationships are not limited to the terms of employment contracts; rather, organizations expect their employees to go beyond their formal job descriptions (Hu et al., 2014). In contrast, Western countries are more individualistic, and independence, autonomy, and self-esteem are highly encouraged.

There exist quid pro quo relationships between Western organizations and their members; employees are expected to fulfill their contractual obligations and to perform their work as specified in their job descriptions (Hu et al., 2014). Workers in Uzbekistan tend to be driven by an extrinsic motivation for social approval, precisely to fulfill the expectations of work team and organization, whereas Western employees work hard because they tend to be driven by individually oriented motivations in expectation of fulfilling their needs for personal growth (Hu et al., 2014). By extending the research in a new context, we here establish the external validity of this relationship. Although engagement is deemed practically vital, little attention has been given to how the elements of Kahn’s psychological conditions theory contribute to employee work engagement followed by work output (Christian et al., 2011; Rothbard and Patil, 2011). Applying Kahn’s theory (1990, 1992) as a theoretical framework, we investigated the relationships among job meaningfulness, engagement, task interdependence, and performance.

According to Kahn’s theory (1990, 1992), meaningfulness describes how valuable a work goal is in relation to an individual’s own standards. Employees who have faith that a given work role activity is personally meaningful are likely to fully immerse themselves in it. Engaged individuals experience high connectivity with their work tasks and strive toward task-related goals that are intertwined with their in-role definitions and scripts; they also make extra efforts to resolve job-related problems, which in return leads to high job performance (Christian et al., 2011; Al-dalahmeh et al., 2018). Thus, this study is the first examination of engagement as a mediator in the relationship between job meaningfulness and performance. Meanwhile, supportive, trustworthy coworker relationships produce high work engagement as well (Kahn, 1990), and task interdependence generates positive coworker relationships (Lee et al., 2018). When employees fail to experience meaning in their work, highly interdependent workers provide each other with information, advice, help, and resources, which serve to amplify their work-related attitudes and behaviors (Kim and Oh, 2020).

This study makes several contributions to the literature. First, we respond to calls for attention to the key drivers of work engagement and work outcomes of job meaningfulness in public organizations by studying the influence of job meaningfulness on employee engagement in the Uzbek public sector. We also respond to another call for attention to the potentially varying situational drivers of work engagement (Shuck et al., 2011) by testing the function of task interdependence as a moderating factor in the relationship between job meaningfulness and work engagement. We examine task interdependence as a possible boundary condition that increases the importance of job meaningfulness for work engagement, postulating that the power of job meaningfulness to positively affect work engagement is stronger for employees with low task interdependence and weaker for those with high interdependence.

**FIGURE 1:**
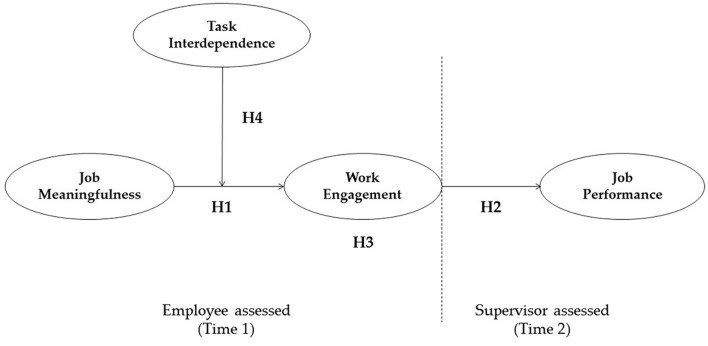
Framework of research model.

Second, we aimed with to analyze the direct links between engagement and performance in a non-Western context, namely, in Uzbekistan. By doing so, we validated the previously known relationships between the study variables in a new context. Replication studies play an important role in the social sciences (King, 2011). Study findings should be continuously revalidated in new work contexts to provide evidence of their generalizability (Mackey and Porte, 2012). Third, we anticipated with this study that the extent to which employees value their work as meaningful will play an important role in facilitating job performance through work engagement. Researchers have separately tested the relationships between job meaningfulness and work engagement (Demirtas et al., 2017; Mostafa and Abed El-Motalib, 2020) and between work engagement and performance (Buil et al., 2019; Ismail et al., 2019), but for this study, we integrated three constructs in one model. With the model, we examined work engagement as an intermediary mechanism through which employees’ perceptions of job meaningfulness affect performance. Above all, the propositions of Kahn’s model have rarely undergone empirical scrutiny (Christian et al., 2011), and here, we apply Kahn’s theory to better explain the relationships between study variables, which will contribute to this line of research. The proposed research model is depicted in Figure 1. The article is structured as follows. First, we discuss the direct relationships among study variables, followed by giving overviews of the mediating role of work engagement in the relationship between meaningfulness and performance and of the moderating role of task interdependence on the relationship between meaningfulness and engagement in the literature review. Following that, we describe the research methodology and present the results of ordinary least squares regression-based analysis and bootstrapping. Finally, we discuss the implications of the study’s findings for both theory and practice.

## Literature Review and Hypotheses Development

### Job Meaningfulness and Work Engagement

Arnold et al. (2007) described meaningful work as “finding the purpose in work that is greater than the extrinsic outcome of the work” (p. 195). Job meaningfulness relates to the extent to which an individual employee derives positive meaning from work (Ahonen et al., 2018), and it involves the fit between work and different domains of the self (i.e., values, beliefs, and norms) (Rosso et al., 2010). Kim and Beehr (2018) consider that employees experience meaningful jobs when they integrate their efforts with the organizational goals and provide valued services or goods that help their coworkers and their organizations to develop.

Job meaningfulness has been indicated to have three dimensions: significance, broader purpose, and self-realization (Martela and Pessi, 2018), where self-realization refers to fulfilling needs, desires, and motivations associated with self-actualization; self-actualization refers to the extent to which employees are able to realize and express themselves through their work. Finding broader purpose of work is related to the belief that the work contributes to the greater good rather than to personal gain and makes the world a better place. Significance is germane to the intrinsic value people find in their work or assign to their work.

Work engagement refers to high personal investment in one’s work role and includes the characteristics of being energized, cognitively vigilant, and willing to invest extra effort to achieve goals (Sonnentag et al., 2010). Research interest in work engagement has increased in recent decades; currently, it is an extremely relevant and meaningful area of inquiry (Karatepe and Karadas, 2015). Work engagement is a construct comprises three dimensions: vigor, dedication, and absorption (Schaufeli et al., 2002b). Schaufeli et al. (2002b) define vigor as expressions of high energy and motivation at work and dedication as indicating an employee’s perceptions of the meaning of work; dedication entails a sense of pride in the job and its challenges. Finally, absorption refers to the degree to which an employee is focused on and happily engrossed in work; absorbed workers are unaware of the passage of time and find it difficult to detach themselves from their tasks (Schaufeli et al., 2002b).

Several researchers have underpinned the importance of work engagement in organizational development (Demerouti et al., 2010; Kim and Park, 2017). Although organizations have tremendous interest in engaged workers, there has been only very limited attention on studying the antecedents of work engagement in public sector organizations (Andrews and Mostafa, 2019; Mostafa and Abed El-Motalib, 2020). Therefore, in this study, we propose job meaningfulness as an indispensable driver of work engagement in public organizations.

Meaningfulness is an important job resource (Fairlie, 2011) that might crucially influence work engagement (Ahmed et al., 2019). Kahn (1990) posited job meaningfulness as a critical psychological condition of engagement. According to Kahn’s psychological conditions theory (1990, 1992), employees drive their personal energies into role behaviors (self-employment) and display the self within the role (self-expression) when three psychological conditions are met: psychological safety, meaningfulness, and availability. According to the theory, employees ask themselves three questions: (1) To what extend is my job meaningful enough to bring myself into this performance? (2) How safe is it to do so? (3) How available am I to do so? The answers to these questions tend to dictate the levels of personal engagement. Kahn (1990, 1992) explained that when employees believe their work is worthwhile and meaningful enough to add value and significance to accomplishments at work, they bring their physical, cognitive, and emotional selves into this work, exhibiting engagement.

Meaningfulness is believed to satisfy psychological needs for purposefulness and belongingness, which further promotes work engagement (Wang and Xu, 2019). Researchers have consistently linked job meaningfulness to increased work motivation, which leads to higher work engagement (Aryee et al., 2012). In addition, people who report having meaningful work are motivated to invest more of themselves in their work role (i.e., engagement) because they feel that in doing so, they will be better able to protect and enhance their well-being (Fletcher, 2019). Macey et al. (2011) argued that “[p]eople come to work for pay but get engaged at work because the work they do is meaningful” (p. 69). In line with this, the perception of job meaningfulness “fuels the motivation to make a prosocial difference that in turn increases effort and persistence” (Sonnentag, 2017, p. 15). Indeed, researchers have associated job meaningfulness with career variables that reflect dedication to one’s career and a passion to put in extra effort (Steger et al., 2012). When employees perceive their work as meaningful, they are more energized and ready to sacrifice their time in pursuit of their careers (Bunderson and Thompson, 2009).

In support of the positive relationship between job meaningfulness and work engagement, Stairs and Galpin (2010) found that employees working in jobs that they perceive as personally meaningful tend to be more engaged than those who are not. Indeed, growing evidence demonstrates a positive association between job meaningfulness and work engagement (Aryee et al., 2012; Demirtas et al., 2017; Fletcher, 2019; Mostafa and Abed El-Motalib, 2020). Based on these findings, we postulate the following hypothesis:

*Hypothesis 1:* Job meaningfulness positively relates to work engagement.

### Work Engagement and Performance

The effects of work engagement on job performance as a predictor have been an increasing topic of academic study (Demerouti et al., 2010). Demerouti et al. (2010) and Kim and Park (2020) highlighted many advantages to employee engagement. Engaged employees exhibit high energy and strong mental resilience, and they tend to voluntarily invest considerable effort in their assigned tasks. Moreover, highly engaged employees tend to have a sense of their work’s significance and challenges, and they express enthusiasm and pride in their work, thus enhancing their performance. Although the concept of engagement is popular, and the number of studies in the engagement-performance domain is increasing (Demerouti et al., 2010), the subject has not received adequate research attention in non-Western contexts (Ismail et al., 2019). Based on this limitation, we aimed with this study to test the direct link between employee engagement and performance in a non-Western, Muslim context, namely, Uzbekistan.

Kahn (1990, 1992) posited work engagement as a psychological state of mind whereby people are attentive, connected, integrated, and focused in their role performance and stated that employees’ “being there” gives them access to their considerable energies and talents in fulfilling work-related tasks and goals. Many studies have shown a significantly positive relationship between employee engagement and performance (Ismail et al., 2019; Zheng et al., 2020). Kahn (1990, 1992) further stated that work engagement refers to a psychological connection with performing work tasks rather than attitudes toward the job itself. Engaged individuals approach tasks with a sense of self-investment, energy, and passion, which should translate into higher in-role and extra-role performance (Kahn, 1990, 1992). Moreover, engaged employees are excited about their work (Bakker, 2009), and enthusiastic employees are positively driven to perform better at work.

Bakker (2009) determined that because engaged employees experience positive emotions (e.g., happiness, joy, enthusiasm), possess psychological and physical health, create their own job and personal resources (e.g., support from others), and are willing to transfer their engagement to others, they perform better than do non-engaged workers. Recent researchers have also found that engaged employees are full of energy and have abundant resources (Demerouti et al., 2015; Scafuri Kovalchuk et al., 2019), and employees who use these resources to cope with job demands have better job performance (Bakker et al., 2011). Similarly, Rich et al. (2010) established that cognitive, emotional, and physical resources are indispensable to employees’ abilities to contribute to organizational goals. In line with this finding, employees who are energized and dedicated to their work have higher intrinsic motivation because their psychological needs (i.e., autonomy and competence) are being satisfied (Wu and Lee, 2020); this satisfaction then further facilitates increased work output. Employees who are partial about their work (Schaufeli and Bakker, 2004) strive to improve their work and establish better work environments (Bakker, 2011). That is why work engagement is such a strong influence on organizational performance; it has been empirically associated with job satisfaction, organizational commitment, organizational citizenship behavior, and knowledge sharing (Dajani and Zaki, 2015; Bailey et al., 2017; Al-dalahmeh et al., 2018; Orgambídez et al., 2019; Yan et al., 2019; Wu and Lee, 2020).

Schaufeli et al. (2006b) conducted two studies on the relationship between work engagement and job performance. Schaufeli et al. (2006b) conducted a cross-sectional study on a large and heterogeneous sample of Dutch employees and identified positive associations between work engagement and in-role performance (*r* = 0.37), extra-role performance (*r* = 0.32), and innovativeness (*r* = 0.37). Schaufeli et al. (2002a) had previously studied the influence of work engagement in the education context in a group of Dutch, Spanish, and Portuguese students; the authors found that work engagement was an antecedent of academic performance. Bakker and Bal (2010) noted that work engagement had a positive influence on job performance in the finding that supervisors rated engaged employees highly on in-role and extra-role performance; engaged employees performed well and were willing to engage in discretionary behavior. Considering the above-described findings, we proposed the following hypothesis:

*Hypothesis 2:* Work engagement positively relates to employee performance.

### Work Engagement as a Mediator in the Relationship Between Job Meaningfulness and Performance

The above discussion, in which we examine the constructs separately and demonstrate relationships between job meaningfulness and work engagement on the one hand and between work engagement and job performance on the other, implies that work engagement plays a mediating role between job meaningfulness and performance. Based on Kahn’s psychological theory (1990, 1992), we argue that a sense that work is meaningful induces employees to put their personal energies into role behaviors; they demonstrate the self within the role, which then intensifies their work-related attitudes (i.e., work engagement), thereby improving employee work outcomes, particularly job performance. Engaged employees are more attentive and focused on responsibilities, are committed to the tasks pertaining to their roles, and work with greater enthusiasm (Rich et al., 2010; Wang and Xu, 2019).

As individuals find deep meaning in their work, they make substantial investments in their work, place high esteem on the assets they have invested, perceive a strong fit between themselves and their jobs, and experience purpose in their work (Spreitzer, 1995). As a result, individuals who find their work to be highly meaningful feel a deeper sense of engagement (Aryee et al., 2012), which, in turn, yields maximum job performance (Bakker and Bal, 2010). Researchers have also found that performing meaningful work makes employment richer, more satisfying, and more productive (Steger et al., 2013). Research on the dimensions of empowerment has shown meaningfulness to be the strongest predictor of employee work outcomes (Liden et al., 2000).

We also believe that employees who have already assigned intrinsic value to their jobs find their work worth doing, feel enthusiasm toward addressing potential issues and problems at work, and are highly engaged in integrating different perspectives to come up with innovative ideas for increasing their output (Walumbwa et al., 2019). Moreover, when employees feel that their work makes a great contribution to others, they are more committed and give more of their energy to work, which in turn enhances their work output (Pradhan and Pradhan, 2016; Al-dalahmeh et al., 2018). Byrne (2014) determined that when employees feel they are contributing to their work units and organizations through their work, they will be physically and emotionally adept at ensuring that their customers are satisfied, happy, and provided with the highest quality of service. Job meaningfulness allows employees to realize their idealized selves (Demerouti et al., 2015) and satisfies their personal needs (Martela et al., 2018), and this in turn strengthens their motivation to work (Oh and Roh, 2019; Shellhouse et al., 2019). Employees then feel greater job satisfaction (Qi et al., 2020) and identify more closely with their organizations (Mostafa and Abed El-Motalib, 2020), which thus enhances their work performance (Inuwa, 2016; Yuen et al., 2018; Miao et al., 2019). Accordingly, the following hypothesis is derived:

*Hypothesis 3*: Work engagement mediates the relationship between job meaningfulness and performance.

### Task Interdependence as an Interacting Variable in the Relationship Between Job Meaningfulness and Work Engagement

In this study, we suggest that job meaningfulness and task interdependence have an interactive effect on work engagement. Specifically, for employees, finding their work meaningful has a strong influence on work engagement when task interdependence is low, where task interdependence refers to the extent to which employees must work together to complete their jobs (Pinjani and Palvia, 2013). Because task interdependence is likely to be linked with relational energy provided by social interactions in groups that enhance task and role capacity (Lee et al., 2017), employees in environments with low task interdependence must perform and complete their tasks individually, which hinders cooperation and group cohesion and decreases employees’ confidence and motivation; this in turn is followed by less work engagement (Rothbard and Patil, 2011).

In that case, employees who feel meaning in their work are more likely to weather the absence of task interdependence to drive work engagement in employees. Because employees with a strong sense of job meaningfulness have high positive energy for work, are willing to learn in anticipation of personal growth, and are highly likely to make large contributions to their organizations by fully engaging with their work (Ghadi et al., 2013), these employees might not need task interdependence to be fully engaged in work. The role of interdependence might not be as important to them as it is for employees who lack job meaningfulness.

In contrast, employees on highly interdependent teams interact more and in turn establish close relationships (Lee et al., 2017; Kipkosgei et al., 2020). Kahn found rewarding interactions with coworkers to be the foundation for increased work engagement (Kahn, 1990; Rothbard and Patil, 2011). A negotiable relationship tends to drive personal energies into role behaviors and demonstrate the self within the roles (Kahn, 1990, 1992), and social characteristics tend to reveal resilience and security in employees (Christian et al., 2011; Rothbard and Patil, 2011). Study findings confirm that supportive coworker relationships play a considerable part in employees’ work engagement (Gullahorn, 1952; Loehr et al., 2005) and performance (Khusanova et al., 2019; Opoku et al., 2019). Employees with harmonious relationships with their coworkers tend to feel more secure about sharing their true selves with others at work, which tends to strengthen attachments in work settings (Avery et al., 2007). Thus, employees working interdependently fulfill their need for relatedness which in turn enhances their work engagement (Lee et al., 2017).

Kahn (1990, 1992) placed much emphasis on psychological experiences of work and work contexts that shape how people present and absent their selves during task performance. Thus, the absence of one condition such as meaningfulness might hinder employees from exposing the “self” at work (Kahn, 1992). We maintain that task interdependence as a situational factor can substitute the effect of meaningfulness on work engagement. Our assumption is that an interdependent work environment where individuals depend on one another to implement tasks is more likely to foster camaraderie and better relationships among coworkers and thereby produce more positive responses and energies at work. This, in turn, will drive employees’ increased attachment to their work.

We believe that when employees fail to find meaning in work, an interdependent working environment obtains the desired levels of employee work engagement. Task interdependence exerts a similar effect on engagement to that of meaningfulness and thereby replaces it. Considering the above-described findings, we postulate the following hypothesis:

*Hypothesis 4:* Task interdependence has an interaction effect on the relationship between job meaningfulness and work engagement such that the relationship is stronger when task interdependence is low than when it is high.

## Materials and Methods

### Sample and Procedure

For this study’s survey, we recruited employees from Uzbek public sector organizations using convenience sampling. The organizations operated in diverse industries: education, utilities, finance, and construction. Before the survey, organization managers were emailed a letter of request regarding the study and its purposes to obtain permission to conduct the survey along with the supervisor’s letter confirming the confidentiality of all collected data. After permission was granted, research assistants distributed the questionnaires to the prospective respondents, explained the purpose of the study, and informed them that all their responses would be private and confidential. The respondents were asked to carefully read each statement on the questionnaire and give truthful responses, and all were instructed to seal and return their completed questionnaires using the return envelopes provided. Using the employee identification lists provided by the human resources departments, the research assistants coded the questionnaires to match the employees to their supervisors. The sample consisted of school and vocational college teachers (50%), employees of utility organizations (22%), an architecture firm (21%), and a national bank (7%).

Because of the possibility of common method bias (Podsakoff et al., 2012), we administered the survey in two stages to two groups of respondents: employees and supervisors. In Stage 1, we collected data from the employees regarding individual job meaningfulness, work engagement, task interdependence, and personal characteristics. In Stage 2, we asked the 47 supervisors to evaluate their employees’ job performance. Altogether, we distributed 307 questionnaires and obtained 183 valid questionnaires in response (response rate: 60%). Among the focal employee respondents, 60% were female, and the average age was 33.39 (*SD* = 8.27). Most of the sample (30%) was between 25 and 30 years old, followed by the 31- to 36-year-old group at 27%. The employee respondents’ mean organizational tenure was 5.88 years (*SD* = 4.79), and 47% of respondents had a bachelor’s degree, whereas 32% of the subjects had a lyceum or vocational college degree. Table 1 presents a summary of the sample description.

**TABLE 1:** Survey respondents’ personal characteristics.

Personal characteristics	Description	No. (%)
Sex	Male	73(39.89%)
	Female	110(60.11%)
Age	19–24	24(13.11%)
	25–30	54(29.51%)
	31–36	50(27.33%)
	37–42	27(14.76%)
	43–48	18(9.84%)
	49–54	7(3.83%)
	55–60	3(1.65%)
Education	Lyceum or college	59(32.24%)
	Bachelor’s degree	86(46.99%)
	Master’s degree	38(20.77%)
Organizational tenure	<1	29(15.84%)
	1–3	49(26.78%)
	4–6	30(16.4%)
	7–10	41(22.42%)
	11–15	28(15.31%)
	16–20	6(3.29%)
Industry	Education	91(49.73%)
	Utilities	41(22.40%)
	Construction	39(21.31%)
	Finance	12(6.56%)

*n = 183. Organizational tenure and age in years.*

### Measures

All questionnaire items were rated on five-point Likert-type scales that ranged from 1 = *strongly disagree* to 5 = *strongly agree*. We translated questions written in English into Uzbek and Russian using standard translation and back translation (Brislin, 1980) to ensure the reliability and validity of the research instrument (all of the questionnaire items are listed in the Appendix).

#### Job Meaningfulness

We employed Spreitzer (1995) three-item scale of job meaningfulness to assess the extent to which the employee respondents found meaning in their jobs (α = 0.78; sample item: “The work I do is very important to me”). The three items measuring job meaningfulness had factor loadings ranging from 0.67 to 0.76.

#### Work Engagement

We measured the employee respondents’ work engagement using Schaufeli et al. (2006a) nine-item scale (α = 0.90; sample item: “At my work, I feel bursting with energy”). The nine items’ factor loadings ranged from 0.54 to 0.80.

#### Task Interdependence

We used Campion et al.’s (1993) three-item scale (α = 0.70) to measure the employee respondents’ task interdependence (sample item: “I cannot accomplish my tasks without information or materials from other members of my team.” The factor loading values ranged from 0.53 to 0.63.

#### Job Performance

The supervisor respondents rated the employee respondents’ job performances using the three-item scale (α = 0.82) developed by MacKenzie et al. (1993; sample item: “This employee is one of my best agents”). The three items measuring job performance had factor loadings ranging from 0.71 to 0.76.

#### Personal Characteristics

The employee respondents provided information about age, gender, educational attainment, and current organizational tenure.

#### Control Variables

Researchers found a positive relationship between employees’ work experience, gender, and work-related attitudes and behaviors (Ismail et al., 2019; Opoku et al., 2020). In addition, highly educated employees were more likely than their less educated counterparts to effectively contribute to work-related activities and demonstrate better performance (Wulandari, 2017). Therefore, we included the employee respondents’ gender, educational attainment, and organizational tenure as control variables in the analyses. Age and organizational tenure were measured in years, and the following were the options for educational attainment: upper-secondary school, 3-year college or lyceum, bachelor’s degree, master’s degree, and PhD. For gender, 0 = male and 1 = female.

### Analytical Approach

We conducted all analyses using STATA 14.2 statistical software. Before testing the hypotheses, we performed a confirmatory factor analysis to examine the distinctiveness of the study variables and generated chi-square statistics and the RMSEA, CFI, and TLI goodness-of-fit indices. To test the hypothesized direct relationships and interaction effects, we performed a series of stepwise regression analyses. First, we regressed the set of control variables (gender, educational attainment, and organizational tenure) on work engagement (Step 1). We added job meaningfulness and task interdependence as independent variables in Step 2, and in Step 3, we included the interaction term between job meaningfulness and task interdependence. We then regressed the control variables on job performance (Step 4), followed by job meaningfulness, work engagement, and task interdependence (Step 5).

To create the interaction term, we mean-centered job meaningfulness and task interdependence before creating the product term. We tested the statistical significance of the indirect effect of job meaningfulness on job performance through work engagement using bootstrapped resampling procedure of 5,000 bootstrapped resamples. Bootstrapping is a computationally intensive method involving repeated sampling from the data set and estimating the indirect effect in each resampled data set (Preacher and Hayes, 2008). Under most circumstances, bootstrapping is the most powerful and reasonable approach to generating confidence limits for specific indirect effects. We calculated 95% bias-corrected confidence intervals (CIs) to determine whether the proposed mediating variable, work engagement, helped explain the relationship between job meaningfulness and performance (Preacher and Hayes, 2008). In this study, we obtained the 95% CI of the indirect effect with 5000 bootstrapped resamples. There is a significant indirect effect through the mediator between dependent and independent variables if the 95% CI does not contain zero. We also performed structural equation modeling (SEM) to test all of the hypothetical relationships as an additional check.

## Results

### Descriptive Statistics

Table 2 presents the means, standard deviations, and correlations between variables and indicates that work engagement was significantly correlated with job meaningfulness and job performance. There was also a significant correlation between gender and job performance. Before running regression analyses, we checked for any possible multicollinearity threats in our data. Table 2 shows that the correlation coefficients between the predictor variables were below the recommended cut point of 0.70 (Ismail et al., 2019). Moreover, we calculated a variance inflation factor (VIF) for every variable, including the interaction terms, and all VIFs were smaller than 10 (Chatterjee and Hadi, 2015), indicating no multicollinearity.

**TABLE 2:** Means, standard deviations, and correlations between variables.

Variable	Mean	SD	1	2	3	4	5	6	
1. Gender	0.60	0.49							
2. Education	2.89	0.72	−0.16*						
3. Tenure	5.88	4.79	0.18*	0.06					
4. Work engagement	3.94	0.57	0.08	0.03	–0.13	(0.90)			
5. Job meaningfulness	4.10	0.55	0.12	–0.01	0.01	0.61**	(0.78)		
6. Job performance	3.95	0.54	0.23**	–0.06	0.03	0.22**	0.07	(0.82)	
7. Task interdependence	3.73	0.66	–0.03	–0.09	–0.09	0.14	0.05	0.09	(0.70)

n = 183; *p < 0.05; **p < 0.01. Tenure = Organizational tenure in years. Regarding gender, 0 = male and 1 = female. Regarding educational attainment, 1 = upper-secondary school, 2 = lyceum or vocational college, 3 = bachelor’s degree, 4 = master’s degree, and 5 = PhD (doctoral) degree. Cronbach’s alpha values are reported in the diagonal.

### Measurement Model

Table 3 presents the model fit statistics of the measurement models. As shown, the baseline four-factor model (χ^2^ = 170.125, df = 124; RMSEA = 0.05; CFI = 0.97, and TLI = 0.96) was a better fit than the three-, two-, and one-factor models, providing evidence of the construct distinctiveness of job meaningfulness, work engagement, task interdependence, and job performance.

**TABLE 3:** Chi-square difference tests among alternative measurement models.

Model	χ^2^	df	CFI	TLI	RMSEA	Δdf	Δχ^2^
4-factor model (hypothesized model)	170.125**	124	0.97	0.96	0.05	–	–
3-factor model (JM and WE merged)	224.125***	127	0.93	0.92	0.07	3	54***
2-factor model (JM, WE, and TI merged)	306.427***	129	0.87	0.85	0.09	5	136.302***
1-factor model (all variables merged)	487.893***	130	0.75	0.70	0.12	6	317.768***

n = 183; **p < 0.01; ***p < 0.001. JM, job meaningfulness; WE, work engagement; TI, task interdependence; CFI, comparative fit index; TLI, Tucker–Lewis index; RMSEA, root mean square error of approximation.

### Hypotheses Testing

To test Hypotheses 1 and 2 relating to engagement and performance as outcome variables, we performed stepwise regression analysis. We entered the control variables in Model 1 (Table 4), which explained 3% of the variance in work engagement. Model 2 (40% of the variance in work engagement) involved testing the first hypothesis, which postulated a positive relationship between job meaningfulness and work engagement. The Model 2 findings (Table 4) indicate that the employee respondents’ perceptions of job meaningfulness were positively and significantly related to their work engagement (*b* = 0.62, *SE* = 0.10, *p* < 0.001), supporting Hypothesis 1. Model 4, which included the control variables, explained 5% of the variance in predicting employee performance, whereas Model 5 with 11% of the variance demonstrated the second hypothesis results. In Hypothesis 2, we assumed that employees’ perceptions of work engagement would be positively associated with job performance, and the regression results (Model 5, Table 4) demonstrate support for the hypothesis 2 (*b* = 0.26, *SE* = 0.09, *p* < 0.01).

**TABLE 4:** Hierarchical multiple regression results for work engagement and job performance.

Variables	WE			JP	
	M1	M2	M3	M4	M5
Intercept	3.84***	3.87***	3.87***	3.85***	3.84***
	(0.22)	(0.17)	(0.16)	(0.17)	(0.18)
Gender	0.13	0.05	0.01	0.25***	0.24**
	(0.09)	(0.07)	(0.06)	(0.08)	(0.08)
Education	0.05	0.05	0.05	–0.01	–0.02
	(0.07)	(0.05)	(0.05)	(0.05)	(0.06)
Tenure	−0.02*	−0.02**	−0.01*	–0.00	0.00
	(0.01)	(0.01)	(0.01)	(0.01)	(0.01)
JM		0.62***	0.59***		–0.12
		(0.10)	(0.10)		(0.09)
TI		0.09	0.16*		0.06
		(0.06)	(0.07)		(0.06)
JM × TI			−0.30*		
			(0.13)		
WE					0.26**
					(0.09)
*R* ^2^	0.03	0.40	0.43	0.05	0.11
*F*	2.19	11.16***	11.73***	3.87*	3.61**
Δ*R*^2^		0.37	0.03		0.06

n = 183; *p < 0.05; **p < 0.01; ***p < 0.001. WE, work engagement; JM, job meaningfulness; JP, job performance; TI, task interdependence. Model reflects unstandardized regression coefficients with standard errors in parentheses.

To assess Hypothesis 3, we tested the indirect effect of job meaningfulness on job performance via work engagement using bootstrapped mediation with 5,000 repeated resamples and percentile bootstrapped CIs. The results presented in Table 5 confirm the indirect effect of job meaningfulness on job performance through work engagement (*b* = 0.17, *SE* = 0.06; 95% CI = [0.06, 0.31]), as indicated by the fact that no CIs equaled zero. This finding supports Hypothesis 3.

**TABLE 5:** Mediating effect of work engagement.

Indirect effect	Estimate	SE	95% CI
JM →WE →JP	0.17	0.06	[0.06, 0.31]

*n = 183. JM, job meaningfulness; WE, work engagement; JP, job performance; SE, standard error; CI, confidence interval.*

**FIGURE 2:**
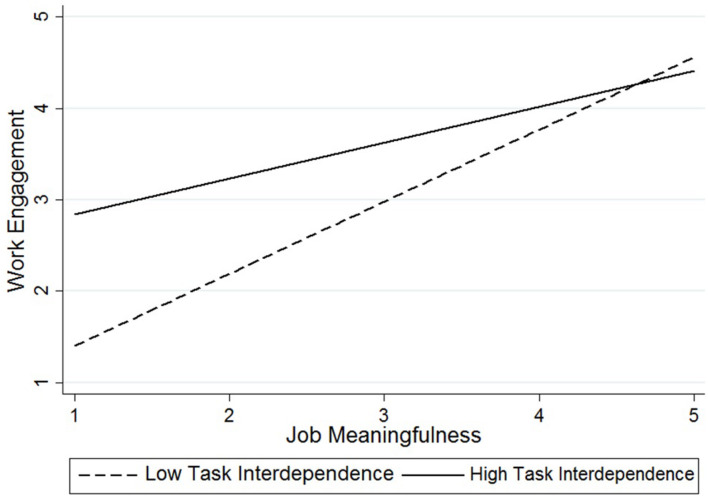
Interaction effect between job meaningfulness and task interdependence for employee work engagement.

Hypothesis 4 predicted that task interdependence moderated the relationship between job meaningfulness and work engagement such that the relationship was stronger for employees with low task interdependence. The results shown in Model 3 (Table 4) support Hypothesis 4 based on the significant interaction effect between job meaningfulness and task interdependence (*b* = −0.30, *SE* = 0.13, *p* < 0.05). To further interpret the interaction effect, we conducted a simple slope analysis following Aiken and West (1991). Figure 2 shows the moderation effect of task interdependence on the relationship between job meaningfulness and work engagement, which demonstrates that job meaningfulness was related to work engagement. The slope decreased by a larger margin from low to high, that is, from *b* = 0.79 to *b* = 0.39; meanwhile, whereas the statistical significance decreased from *p* < 0.001 at low task interdependence to *p* < 0.05 at high interdependence. These results provide evidence of the interaction effect, thus supporting Hypothesis 4.

As an additional test, we performed to SEM examine direct, indirect, and moderation effects. The results presented in Table 6 confirmed all of our hypotheses.

**TABLE 6:** Results of mediation and moderation tests for structural models.

Hypothesized paths	Path coefficient	SE	*p*-value	Decision
JM → WE	0.62	0.06	0.000	Supported
WE → JP	0.27	0.09	0.001	Supported
JM → WE → JP	0.17	0.06	0.002	Supported
JM × TI	−0.30	0.09	0.001	Supported

*n = 183. JM, job meaningfulness; WE, work engagement; JP, job performance; TI, task interdependence.*

## Discussion

In this study, employing Kahn’s psychological conditions theory (1990, 1992), we sought to investigate the relationships among job meaningfulness, engagement, and performance. We also tested task interdependence as a boundary condition that would promote employee engagement. In line with prior research (Mostafa and Abed El-Motalib, 2020), the study findings revealed that job meaningfulness is positively related to work engagement, confirming job meaningfulness as a “necessary prerequisite” for work engagement (Albrecht, 2013, p. 243), meaning that employees who find their work significant and worthwhile are more likely to be absorbed and dedicated (Demirtas et al., 2017). Nearly all studies on work engagement have been conducted in Western countries, most notably North America and Western Europe, and there has been insufficient replication of data in non-Western contexts (Kim, 2017; Ismail et al., 2019); for instance, Buil et al. (2019) found a direct relationship between employee engagement and performance among hotel employees in Spain. In one of the few examinations of this link within a non-Western context, Mostafa and Abed El-Motalib (2020) provided similar results among employees of an Egyptian public hospital. Consistent with these preceding studies, our study with a sample of employees from Uzbek public organizations confirmed and contributed to the generalizability of the work engagement-employee performance link in the public sector. Moreover, we elucidated the positive impact of job meaningfulness on employee performance through the potential mediating role of work engagement, meaning that individuals who believe their jobs offer intrinsic consider their work worth doing, feel excitement about addressing work-related issues and problems, and are highly driven to integrate different perspectives to come up with innovative ideas, which tends to guarantee enhanced work output (Walumbwa et al., 2019). Our study findings have also confirmed the moderating role of task interdependence on the relationship between meaningfulness and engagement. Specifically, we found that task interdependence assumed greater importance for employees who failed to find their jobs sufficiently meaningful to engage in their work, whereas employees who already found their jobs meaningful might not have required task interdependence to invest extra effort in their jobs.

### Theoretical Contributions

This study contributes to the literature in a number of ways. Most importantly, we framed our study in the context of testing Kahn’s psychological conditions (1990, 1992), which has not received much attention in empirical studies.

There have been recent calls for research on determining a potential antecedent of work engagement in public organizations (Andrews and Mostafa, 2019; Mostafa and Abed El-Motalib, 2020). Meanwhile, because job meaningfulness plays a critical role in most employees’ choice of public organizations to work for, public administration scholars have urged that the importance of meaningfulness in this sector be analyzed as well (Tummers and Knies, 2013). With this study, we sought to address these calls by examining job meaningfulness as a key driver of work engagement in an Uzbek public setting.

Shuck et al. (2011) also reported on the lack of research on situational drivers of work engagement. Filling this gap in the academic literature, we proposed task interdependence as a boundary condition that facilitates employee engagement and empirically confirmed its moderating role in the relationship between job meaningfulness and engagement; specifically, task interdependence compensated for low meaningfulness or acted as a substitute. We also addressed calls for more research on the relationship between work engagement and employee performance in a non-Western context. Moreover, previous researchers had separately examined the relationship between job meaningfulness and engagement (Demirtas et al., 2017; Mostafa and Abed El-Motalib, 2020) and that between engagement and performance (Buil et al., 2019; Ismail et al., 2019). In this study, we integrated three constructs in one model to test work engagement as a mediating variable through which employees’ perceptions of job meaningfulness affect performance. Finally, based on earlier findings that most studies on the influence of engagement on performance are conducted in Western countries (Kim, 2017; Ismail et al., 2019), we analyzed the data from a sample of public organization employees in Uzbekistan. The study’s findings make an important contribution to work engagement by increasing the international breadth of empirical research findings on the engagement-performance link.

### Practical Implications

Public administration practitioners have acknowledged that engagement and commitment are motivational tools to improve civil servants’ and public service outcomes in the face of political constraints that lead to frequent changes in policy, declining growth in investment, and cost-cutting initiatives (Burke and El-Kot, 2010; Ancarani et al., 2018).

The results of our study have several practical implications for managers and their organizations. Because the study findings suggest that increasing job meaningfulness will increase employee engagement in their work, which in turn improves work performance, it is crucial that public sector organizations stimulate employees’ sense of job meaningfulness. This can be achieved through approaches such as (1) drawing employees’ attention to tasks that encourage them to realize themselves; (2) involving employees in making decisions that make their work more impactful and useful to others, including in redesigning jobs; (3) and developing social connections between employees and public sector clients (Jo et al., 2018; Martela and Pessi, 2018).

Furthermore, the study results suggest that task interdependence can encourage work engagement. Managers can assign tasks that require employees to work interdependently to increase employee engagement, especially when routine tasks might engender low job meaningfulness. Organizations can cope with low meaningfulness by creating high interdependence and vice versa.

In addition, managers could employ high-quality leader-worker relationships to enhance employee engagement (Ancarani et al., 2018). Unlike other restrictions in the public sector, leader-member exchange (LMX) is within the control of managers. Supervisors who engage in high-quality LMX give their employees more of their time, more direct information, more emotional support, and more intrinsic rewards such as empowerment (Ancarani et al., 2018). In turn, employees feel motivated to work harder to benefit the manager in reciprocation (Gouldner, 1960). Leaders who promote high-quality leader-worker relationships provide psychological safety that encourages employees to find their work environments safe spaces to express their true selves and actively engage their interest in work tasks (May et al., 2004). Moreover, employees who are parts of high-quality LMX are more optimistic and self-efficacious, and such beliefs are considered to be important predictors of employee engagement (Ancarani et al., 2018).

Finally, public organizations are highly encouraged to create friendly atmospheres where employees support one another. Schaufeli and Bakker (2004) established the importance of coworkers’ supportive relationships as a significant work resource for achieving goals and work engagement. Employees with harmonious coworker relationships feel more secure about sharing their true selves with others at work, which tends to strengthen engagement in work settings (Schaufeli and Bakker, 2004).

### Limitations and Suggestions for Future Studies

This study has several limitations that should be addressed in future research. Initially, although we attempted to reduce common method bias (Podsakoff et al., 2012) using the two-stage approach and by separating the ratings of the employees and the supervisors by 3 weeks, we did collect the data on job meaningfulness (independent variable), task interdependence (moderator), and work engagement (mediator) at the same time, and thus the data remained susceptible to common method bias.

With our study, we empirically demonstrated that job meaningfulness is positively associated with engagement; however, the effect size was not especially high, which suggests that other factors might have an impact on employees’ levels of engagement in public organizations. Thus, public administration scholars might focus on identifying other antecedents of work engagement in this sector. Moreover, we asked supervisors to evaluate their employees’ job performance (i.e., subjective job evaluation), which served as a boundary condition of the study. The study results should be generalized with caution because they might not be applicable in the context of objective job evaluations. Another study limitation concerns the study sample size (*n* = 183). Although the sample size was considered reasonable for regression analysis (Ismail et al., 2019), we suggest that the study be replicated with a larger sample size. Replication should also consider cultural differences, which we avoided with this study involving Muslim respondents. Finally, our sample was homogeneous in terms of mode of employment in that all survey respondents were full-time employees. We assume that the research findings might differ if data are included for part-time workers.

## Conclusion

For this study, based on Kahn’s theory, we examined the role of job meaningfulness in employee performance via work engagement. We hypothesized and empirically showed that meaningfulness was significantly associated with work engagement and that work engagement was positively related to performance in public organizations. We further found that work engagement had a mediating influence that explained the relationship between job meaningfulness and job performance. We also with this study identified another situational driver (i.e., task interdependence) that maintains employee engagement. Despite the potential limitations of this study, these findings contribute to the growing body of research on work engagement and meaningfulness in the public sector.

## Data Availability Statement

The datasets presented in this article are not readily available because “This is because not all authors consented to the disclosure of survey data.” Requests to access the datasets should be directed to S-WK, global7@gachon.ac.kr.

## Author Contributions

RK is the principal researcher and prepared the first draft of the article. S-WK supervised the study and refined the draft into a publishable article. In addition to motivating the publication of this article, SBC added valuable theoretical and methodological insights based on his knowledge and expertise regarding the topic. All authors have read and agreed to the submitted version of the manuscript.

## Conflict of Interest

The authors declare that the research was conducted in the absence of any commercial or financial relationships that could be construed as a potential conflict of interest.

## Publisher’s Note

All claims expressed in this article are solely those of the authors and do not necessarily represent those of their affiliated organizations, or those of the publisher, the editors and the reviewers. Any product that may be evaluated in this article, or claim that may be made by its manufacturer, is not guaranteed or endorsed by the publisher.

## Appendix

**Job Meaningfulness (α = 0.78) (Spreitzer, 1995)**

1. The work I do is very important to me.

2. My job activities are personally meaningful to me.

3. The work I do is meaningful to me.

**Work Engagement (α = 0.90) (Schaufeli et al., 2006a)**

**Vigor**

1. At my work, I feel bursting with energy.

2. When I get up in the morning, I feel like going to work.

3. At my job I feel strong and vigorous.

**Dedication**

4. I am proud of the work that I do.

5. I am enthusiastic about my job.

6. My job inspires me.

**Absorption**

7. I get carried away when I am working.

8. I feel happy when I am working intensely.

9. I am immersed in my work.

**Task Interdependence (α = 0.70) (Campion et al., 1993)**

1. I cannot accomplish my tasks without information or materials from other members of my team.

2. Other members of my team depend on me for information or materials needed to perform their tasks.

3. Within my team, jobs performed by team members are related to one another.

**Job Performance (α = 0.82) (MacKenzie et al., 1993)**

1. This employee is one of my best agents.

2. All things considered, this employee is outstanding.

3. All things considered, this employee performs his/her job the way I like to see it performed.
